# An Integrative Review of How Healthcare Organizations Can Support Hospital Nurses to Thrive at Work

**DOI:** 10.3390/ijerph17238757

**Published:** 2020-11-25

**Authors:** Willoughby Moloney, Jessica Fieldes, Stephen Jacobs

**Affiliations:** 1 School of Nursing, University of Auckland, Auckland 1142, New Zealand; s.jacobs@auckland.ac.nz; 2 Starship Hospital, University of Auckland, Auckland 1023, New Zealand; jfieldes@adhb.govt.nz

**Keywords:** nurses, wellbeing, burnout, thriving, health and safety practices, occupational health, public health, working conditions, workplace policies, prevention

## Abstract

Background: Solutions that address the anticipated nursing shortage should focus on thriving at work: a positive psychological state characterized by a sense of vitality and learning, resulting in higher levels of work engagement, commitment, and wellbeing. Purpose: To synthesize international evidence on organizational factors that support hospital nurse wellbeing and to identify how the Social Embeddedness of Thriving at Work Model can support health managers to develop management approaches that enable nurses to thrive. Method: Conduct an integrative review of literature published between 2005–2019. Results: Thematic analysis identified five key themes: (1) Empowerment; (2) Mood of the organization; (3) An enabling environment; (4) Togetherness with colleagues; and (5) Leaders’ connectivity. Conclusions: The Social Embeddedness of Thriving at Work Model supports managers to develop management approaches that enable their nurses to thrive. Health managers should consider strategies to support nurses to thrive at work to improve nurse work engagement and wellbeing.

## 1. Introduction

Internationally, nursing is facing unsustainable working conditions, high turnover rates, and perceived staff stressors, hindering efficient nursing care and adversely affecting healthcare outcomes [1]. Nurses often have intense and demanding workloads, resulting in them feeling emotionally and physically exhausted [2]. Nurses are at greater risk of injury and physical illness than the general public [3,4] and are at greater risk of mental illness than the general public due to stress, burnout, and psychological disequilibrium [5,6]. A serious nursing shortage is predicted, which will increase demand on those nurses remaining and intensify levels of staff burnout and intention to leave [7,8]. These issues have been heightened with COVID-19 [9,10,11]. Therefore, nurses being resilient against adversity alone is not sufficient. Organizations need to find new ways to support vulnerable staff and prioritize wellbeing [12]. One approach advocated by the field of positive organizational scholarship is ‘thriving at work’, a construct based on learning, vitality, psychological, and behavioral outcomes [13].

Thriving at work is a means to sustainability and organizational effectiveness through healthy, high performing, and committed employees [14]. Nurses are crucial to organizational success within the health system, therefore thriving at work would not only support how they work, reduce absenteeism and improve wellbeing, but also positively impact the patients they care for through improved working conditions, quality of care, and attention to patient safety [7,15,16,17,18].

The Social Embeddedness of Thriving at Work model [13,15] provides a positive organizational scholarship approach to sustainability and organizational effectiveness through developing healthy, high performing, and committed employees [14]. Nurses are crucial to organizational success within the health system, therefore thriving at work would not only support how they work, reduce absenteeism, and improve wellbeing, but also positively impact the patients they care for through improved working conditions, quality of care, and attention to patient safety [7,15,16,17,18].

A significant outcome of thriving at work is engagement, which reflects the degree of emotional and intellectual involvement a person has when embracing work tasks [7,19,20]. Factors that contribute to engagement (across both the business and nursing literature) are participation (or control), reward, community, fairness, workload, and values, all of which can be guided by management [7,21,22]. As nurses face increasing demands, a focus on improving work engagement, and therefore thriving, may result in improved commitment [23,24,25,26,27].

Whittemore and Knafl [28] define the integrative review as a method that includes both experimental and non-experimental research and plays an important role in evidence-based practice for nursing. The methodological approach includes five stages: problem identification; literature search with comprehensive search strategy; data evaluation; data analysis; and synthesizing of findings. The purpose of this literature review was to: (1) explore international evidence on organizational factors that enable the creation of psychologically healthy workplaces that can positively influence the individual health and development (wellbeing) of nurses; (2) explore the evidence about the Social Embeddedness of Thriving at Work Model and its effectiveness, and; (3) then see if there is some common ground between the two and determine whether research to date does support the use of the Thriving at Work model as a way to support health managers to develop management approaches that enable nurses to thrive.

## 2. Methods

### 2.1. Search Strategy

An integrative review of literature published between 2005–2019 was conducted. The utilized databases included the Cumulative Index of Nursing and Allied Health Literature (CINAHL), MedlineOVID, PsycINFO and Proquest ABI/Inform. Hand-searching alongside forward and backward searching of reference lists yielded positive results when searched for in Google Scholar. Key search words included: hospital*, nurs* OR nurses OR nursing, well-being OR wellbeing OR well being, organisation* OR organization*, factor OR characteristic, thrive OR thriving. Keywords were linked by AND and utilized across the databases.

Inclusion and exclusion criteria are outlined in Table 1. As limited literature discussing thriving at work was found within the nursing literature, the inclusion criteria were broadened to include relevant literature within business and psychology databases. The target population was hospital-based nurses, yet the inclusion of business and psychology literature was necessary, as they academically pioneered the emerging concept of thriving at work. Articles were accepted if they described either/or wellbeing and thriving at work. Preference was given to research studies over gray literature.

**Figure 1:**
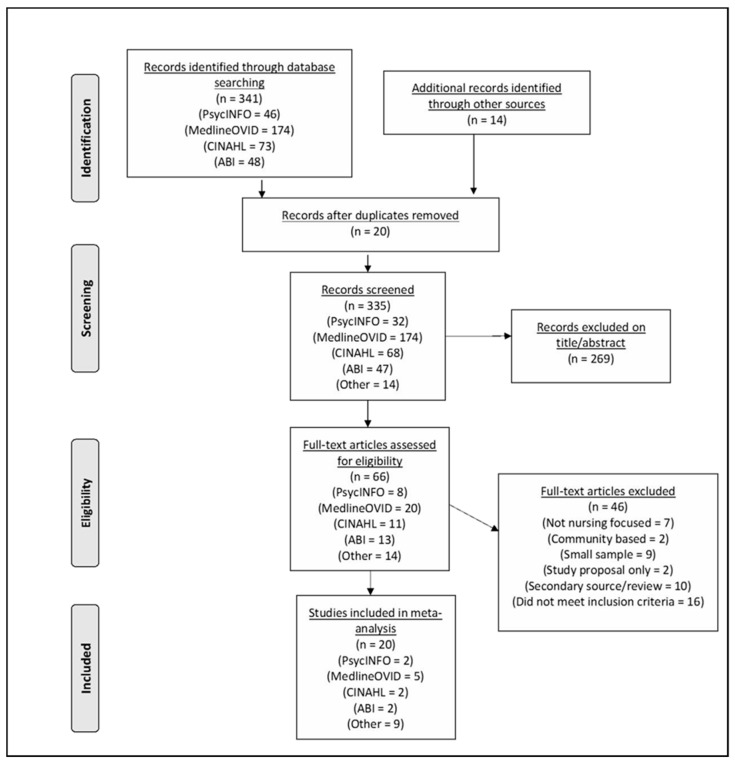
PRISMA flow diagram.

The four-phase PRISMA tool was utilized to summarize the phases of the review and uphold the transparency of the literature search (Figure 1).

The aim of the PRISMA flow diagram is to guide how reviews are conducted, promote quality results, and reduce flawed reporting [29].

A total of 341 articles were relevant for review. Hand searching and forward and backward searching of reference lists then identified a further 14 articles. A total of 20 articles were removed due to duplicity. Afterwards, 335 articles were then screened via titles and abstracts using the inclusion and exclusion criteria. We then excluded 269 articles, leaving 66 articles for further analysis. Full text reviews then resulted in a further 46 articles being excluded due to: not focusing sufficiently on organizational factors or thriving at work; coming from a review or a secondary source; not focusing on nurses in hospitals; small sample sizes; and being study proposals only. Ethics approval was not required for an integrative review.

### 2.2. Analysis

A total of 20 articles were included for analysis (Table 2). The 20 articles were appraised for quality using the John Hopkins Nursing Evidence-Based Practice Model (JHNEBP) and Research Evidence Appraisal form [30]. This tool rated 17 articles as having a strength of evidence at Level 3, and three articles a strength of evidence at Level 5. Of the 20 articles chosen for review, 14 were appraised as high quality, and six were appraised as good quality. Collectively the articles produce consistent results, definitive conclusions, and recommendations, including sufficiently referenced evidence [30].

The majority of articles were cross-sectional, relational, comparative, or descriptive non-experimental studies and utilized volunteers with surveys or questionnaires. Authors discussed steps to alleviate the risk of bias such as using existing reliable and valid scales, performing tests to evaluate the extent of bias and collecting data from multiple sources [5,14,23,25,31]. Four authors also declared participant anonymity to foster honesty with answers and help decrease the degree of bias. Further limitations and their occurrence were: cross-sectional data and competing other factors/moderators demonstrated limits on the evidence to determine causality (*n* = 10); single center study considered concerns with generalizability of results (*n* = 5); cultural beliefs and differences (*n* = 3); size of sample at risk of non-response bias (*n* = 3); and lack of control variables was susceptible to social desirability bias (*n* = 1).

## 3. Results

Five overarching themes were developed from the thematic analysis: empowerment, mood of the organization, enabling environment, togetherness with colleagues, and leaders’ connectivity.

### 3.1. Empowerment

This theme represents an energizing way of working that shares control and eliminates hierarchical thinking between leaders and workers. Structural empowerment, that is, the extent to which a work environment provides access to support, resources, information, and opportunities to learn and grow, has been shown to be an important work-unit characteristic that has positive effects on nurses’ job satisfaction and their ratings of the quality of care they are able to deliver [42]. The categories are: autonomy, decision-making latitude, and motivation.

#### 3.1.1. Autonomy

Autonomy refers to the sense that an individual’s behaviors are self-endorsed. Within one’s job, autonomy has beneficial individual and organizational outcomes, including creativity, reduced turnover intention, and improved job satisfaction [13,40]. Autonomy at work is linked to increasing feelings of vitality and greater work satisfaction because individuals are enabled to master their work in novel ways [39]. Because uncertainty and stressors in the workplace are common for nurses, encouragement of autonomous behaviors is important approach for nursing leaders [25]. Nurses who feel in control when making patient-care decisions feel autonomous, experience lower levels of turnover, have healthier interpersonal relationships, and provide greater quality of care [17].

#### 3.1.2. Decision-Making Latitude

Work environments that foster decision-making discretion enable individuals to feel in control through self-directed, agentic behaviors [13,37]. Active participation in organizational matters and decisions builds new skills, improves learning and vitality, and supports thriving at work [33,36]. The learning dimension of thriving is enhanced through competence, feeling comfortable when taking risks, and ‘smarter’ working in the workplace [40]. For nurses, decision-making latitude is predicted by factors such as information sharing, support, resources, and growth opportunities, which Laschinger and Finegan [25] refer to as ‘power lines’. Decision-making latitude positively impacts a nurse’s incentive to learn, job outcomes, competence, and individual wellbeing [17].

#### 3.1.3. Motivation

Thriving is described as encapsulating the trait of motivation (as energy which directs learning) [23]. When teams are experiencing low work satisfaction, low levels of engagement and poor job outcomes, improving each individual’s work motivation is important [40]. Ensuring continuous education and growth opportunities for employees is a pivotal task for organizations; this motivates and leads them to experience an increase in both energy and learning, which is reciprocated through commitment and obligation [15]. Nurses need to be provided with an environment that allows for ongoing professional development if they are to stay current, motivated, committed, and empowered [25].

### 3.2. Mood of the Organisation

This theme embodies individual and team behaviors that are perceived as beneficial for quality working relationships and a successful organizational climate. The categories are: favorable behaviors, climate of civility and discretion, employee engagement, and organizational justice.

#### 3.2.1. Favorable Behaviors

Common concepts related to favorable behavior are trust, respect, appreciation, and a focus on liaisons with leaders. When leaders show trust in an employee (a reciprocated relationship), there is a direct link with thriving at work [23]. Brown et al. [33] uphold that trusting relationships “can act as a secure base and safe haven for exploration” and ultimately learning and vitality. Leaders showing appreciation for a job well done by their employees reduces stress levels because individuals accurately understand their performance progress towards goals [27] which inspires a positive work climate and builds up confidence [5].

#### 3.2.2. Climate of Civility and Discretion

Civility relates to politeness and consideration of others [15]. Strong relationships between employees and their organization are the foundations for thriving at work [23]. Connectivity and civility within social systems are also stimulating, helping to establish boundaries, enhance resources and drive thriving [37]. Individuals are motivated by role-modeling of good behaviors, helping them to feel confident and have a sense of obligation to reciprocate [37]. Nursing leaders need to be authentic by role-modeling acceptable and encouraged behaviors if they are to create this secure and positive climate for thriving [5].

#### 3.2.3. Employee Engagement

Engagement and thriving can be considered complementary, but one can be engaged in their work but not thriving and vice versa [27]. Kleine et al. [15] proposed that energy (or vitality) is a necessary resource when coping with work demands, resulting in individuals who are more mentally healthy and resilient, yet nurses report moderate energy levels [25]. Energy is related to individuals’ psychological needs of connectivity, relatedness, competence, and autonomy [27]. Promoting nurse engagement is an important strategy for retention and is a predictor of job satisfaction [25].

#### 3.2.4. Organizational Justice

Organizational justice is described as an employee’s perception of fairness and is interrelated with commitment, turnover, wellbeing, and performance [32]. It is also about individuals not acting in self-interest but to norms of the workplace environment that impact employees’ equally [37]. In the nursing context, the work climate is influenced by employees being treated fairly; this has a direct impact on individual work successes, how employees cope with demands, and on patient outcomes [5]. Unfair treatment can lead to emotional exhaustion for nurses [25].

### 3.3. Enabling Environment

Enabling environmental factors support employees/nurses’ physical and mental health so that they can provide quality care. The categories are: job demands/stressors, control/demand, professional development, quality of care, and availability of resources.

#### 3.3.1. Job Demands/Stressors

Work stressors that are energy-draining impede future learning opportunities and negatively impact thriving at work [15]. Thriving is not about reducing the stressors, but having the right resources and settings available at the right time [13]. Time pressures, working with patients, demanding workloads due to inadequate staffing, changes in working hours or schedules, and changes in work location (redeployment) are associated with psychological distress and exhaustion in healthcare workers [34,35,41].

#### 3.3.2. Control/Demand

The Demand-Control-Support Model explains the association between job stress, the work environment and psychological wellbeing [17]. A lack of job control is an organizational factor which predicts burnout in healthcare workers [34]. There is a strong relationship between social support, management procedures, and the ability to control and influence one’s work [35]. This can lead stressed individuals to make irrational choices, put in less effort, and settle for substandard patient care [17].

#### 3.3.3. Professional Development

The thriving at work concept includes forward momentum and self-development [13]. This may include goal setting, requesting and listening to feedback, and engaging in growth and developmental activities [38]. A positive care environment is enabled when professional development and education support the improvement of nurses’ skills, competence, and expertise [15,17] as nurses report perceived feelings of freedom, better interpersonal relationships, and lower levels of turnover [17].

#### 3.3.4. Quality of Care

The nursing practice environment is key to quality of care and patient satisfaction [17]. A stressful environment decreases quality of care, patient satisfaction and patient safety [34]. As Sharif et al. [17] discuss, nurses’ psychological wellbeing is critical and directly related to the quality of patient care delivered. Therefore, managers need to address factors relating to negativity in the workplace, whether it is a lack of support, perceived unfairness, or distress if they are wanting a high standard of care to be delivered [17].

#### 3.3.5. Availability of Resources

Nurses notice whether their organization facilitates or constrains their practice [17] by observing whether their workloads match the resources available to them [34]. Effective and safe working conditions include adequate supervision and adequate resources [13]. An optimal environment is one that supports and empowers nurses to work safely within their full scope of practice [17].

### 3.4. Togetherness with Colleagues

This theme represents the collegial elements and social competencies necessary for individuals, and therefore organizations, to work effectively. The categories include: social support environment, collegial relations, and reciprocity.

#### 3.4.1. Social Support Environment

Strong relationships between peers are necessary for work to be accomplished in meaningful ways [25]. The quality of the relationship or community of staff is a significant predictor of work engagement for hospital staff [34]. However, individuals’ levels of thriving are not static, but fluctuate in response to variations and dynamics within the workplace [39]. Social support and team cohesion at work is a protective factor against psychological distress [34], with meaningful and generative social relations significantly assisting individuals to experience vitality and zest [23,37,40].

#### 3.4.2. Collegial Relations

When employees experience continual support from their colleagues, they participate more in decision-making and behave more collaboratively and as a collective [40]. The Thriving at work model includes the concept of heedful relating, which embodies the connectivity of employees looking out for each other through support, while helping to coordinate each other’s work to achieve system goals [13]. Within the nursing literature, informal relationships between colleagues are consistently linked to empowering behaviors, which enables learning from each other’s different approaches to work tasks [25].

#### 3.4.3. Reciprocity

Team members need to collaborate and fairly contribute if they are to work effectively as a group towards accomplishing shared goals, leading to learning and vitality being experienced and therefore thriving at work [38]. Through information sharing, colleagues enable each other to share grievances, complete innovative tasks and obtain new knowledge for their practice [33]. When colleagues share information throughout the team, it promotes planned coordination and results in individuals who feel more confident and competent in their abilities [27]. For nurses, Laschinger and Finegan [25] view the sharing of information between peers as essential.

### 3.5. Leaders’ Connectivity

The leader/employee relationship, particularly leader behaviors, can positively impact thriving. The categories are: support from superiors, interpersonal relationships between management and staff, and acknowledgment.

#### 3.5.1. Support from Managers

Managerial support is important if employees are to take risks, implement their own ideas and learn from experiences [15,18]. They also need to feel protected from uncivil interactions with management modeling acceptable and kind behaviors [37]. Concern for employees’ wellbeing, promotion of their growth, and information sharing creates a climate in which individuals will be focused, curious, resilient, and have professional freedom [38] and offset intentions to leave [19,27].

#### 3.5.2. Interpersonal Relationships between Management and Staff

When organizational support is positively perceived, it is a predictor of work-related wellbeing, pleasant affective feelings, performance and job satisfaction [17], stimulating collective mindfulness and goodwill [19]. Effective communication is foundational, reducing conflict and helping the management of inequalities between management and nurses [35,37]. Relational leadership (rather than task focused) produces more positive work outcomes and directly influences individual nurses’ work-related wellbeing [18].

#### 3.5.3. Acknowledgment

Nursing leaders need to acknowledge and focus on their employee’s wellbeing by strengthening associated factors such as respect, cooperation, satisfying patient-care and professional development [18]. This promotes role meaningfulness, which in turn enhances vitality and competence [15,17]. Feedback increases thriving, as it enables individuals to appraise their progress, resolve feelings of performance uncertainty, and adjust development goals [27]. Employees see incentives, rewards, and career progression as positive feedback for their work and contribution to the organization, which makes them more likely to engage in further innovation ideas [37].

## 4. Discussion

### 4.1. Thriving at Work and Nursing

**Figure 2:**
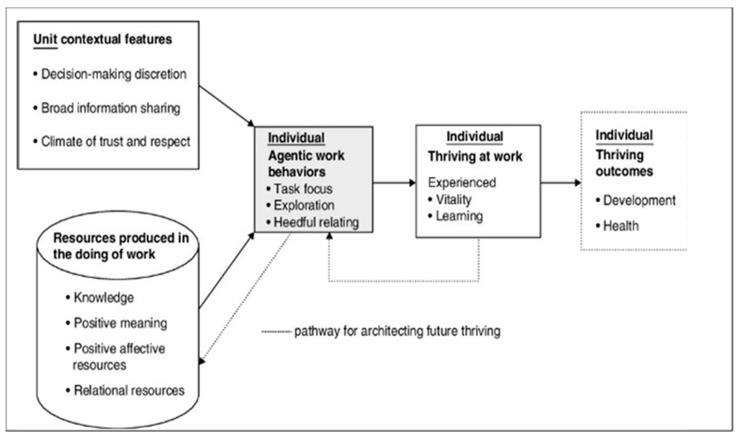
The Social Embeddedness of Thriving at Work Model [13].

The foundational and predominant model focused on thriving in the workplace is the Social Embeddedness of Thriving at Work [13,39] (Figure 2). Nursing is a workforce at risk from factors such as high demand, lack of support, inadequate autonomy, strained collegial relations, a lack of fairness, civility and respect; these all can negatively impact on nurses’ mental-health and job engagement levels [5,16,43,44]. Within the Thriving at Work Model, these factors are represented as threats to individual growth, development, and health [13,44,45].

### 4.2. Unit Contextual Features

#### 4.2.1. Decision-Making Discretion

Decision-making discretion enables nurses to get work done and express new ideas, with a positive impact on feelings of control, incentives to learn and competence [17,25,34]. Leaders should be encouraged to empower problem solving and provide greater decision-making opportunities to those around them [13,37,38].

#### 4.2.2. Broad Information Sharing

When information is readily shared it enables confidence in decision-making and effective responses to situations that arise [27]. Information sharing is as an essential resource for nurses that makes work meaningful and increases feelings of empowerment [25].

#### 4.2.3. Climate of Trust and Respect

Kind and civil interactions foster a climate of trust and respect, resulting in thriving and a clear organizational vision [37,45,46]. A positive organizational climate supports nurses to succeed in their roles and experience constructive outcomes, and good mental health [5,45]. Trust, conflict resolution, and minimizing cynicism from staff is essential for an optimal nursing environment and if workers are to be able to thrive during times of change [25,33,45,46].

### 4.3. Agentic Work Behaviours

#### 4.3.1. Task Focus

Having task focus can provide a sense of learning through refining routines and working more efficiently [13]. Nurses face many everyday distractions (e.g., time pressures and stressful workloads) which may be detrimental to their direction and efficiency [34,47]. Supporting employees to be pro-active, motivated, and to enjoy work will lead to improved thriving at work [15,27,33,40].

##### Exploration

Empowering nurses to work innovatively and creatively enables them to experience increased knowledge, skills, and vitality [13,48]. The curiosity and sharing of ideas will help broaden a healthier working environment for nurses facing increasing demands [22,46].

#### 4.3.2. Heedful Relating

Individuals will support each other to achieve goals in ways that promote affective and psychological energy, resulting in mutual learning [13]. A sense of community and understanding of overall organizational goals among team members helps people create solutions and avoid errors [19], leading to increased belonging and decreased intention to leave [23,33]. Facilitating employees to build empowering relationships (e.g., between nurses, physicians, and colleagues), results in thriving at work with a more positive workplace [5,15,25].

### 4.4. Organizational Factors to Enable Thriving at Work for Frontline Nurses

Nursing leaders are in a privileged position where they can encourage autonomous behaviors, instill confidence, and empower their nurses, which are enablers for thriving at work [25,36,49,50]. The successful movement of ‘leaderful practice’ in healthcare, which promotes free expression, shared engagement, and cooperative efforts, has upended traditional views of leadership [50]. Thriving is not just about individuals self-managing responsibilities and control; it is enabled by the recognition and confidence instilled by leaders, through the sharing of control and encouragement to make decisions freely [36].

A workplace with leaders that endorse team emotional support, respect, and shared goals will boost individuals’ feeling of worth and thriving [27]. An opportunity for nursing support involves the regular utilization of collegial networks of support and debriefing forums to build more resilient teams [43,45,49]. The reciprocating nature of this leader/team relationship is important to promote if behaviors are to be focused and if the organization culture is to improve and accomplish meaningful work and thus thrive [19,43,49].

The positive relationship between thriving, professional development, and nursing is clear [15]. When considering the essential learning component of thriving, nurses would benefit through continually gaining new knowledge, as in the context of thriving, this is also thought to set up individuals to better cope when under hardship (e.g., job demands and times of poor control) and “subsequently thrive, rather than just survive” [33]. Nurses should be provided with a balance between clinical duties and educational opportunities if they are to stay competent and committed and from a thriving at work point of view, nurses would be experiencing learning (growth) and vitality (motivation) [25,37].

Reciprocal trust is a crucial concept for thriving at work [23,24,33,48] Nursing leaders should consider the expression of trust to the employee as a means of showing worthy recognition [23]. Trust is a willingness to express vulnerability, so nurses also need to convey and reciprocate trust in their leader if they are to completely engage [24,48].

Organizational justice plays a significant role in thriving at work and the known link between nurses experiencing unfairness in the workplace and therefore emotional exhaustion deserves attention as a potential barrier to thriving at work [5,25,37]. Management should anticipate that volatile and uncivil interactions are job stressors that will cumulatively impact performance, wellbeing, workplace attitudes and hinder thriving at work [15,16,23,37,51]. For nurses, they must perceive fairness in how they are treated if they are to provide quality care, cope with demands, and avoid emotional exhaustion [51].

Nursing leaders play a critical role when it comes to advocating for a healthy working environment, with job engagement with nursing workloads being a leading contributor [7,19,52]. One focus for healthcare organizations should be on workloads that are balanced with the resources available, as burdensome and disproportionate nursing workloads are unsafe, unhealthy, and hamper quality of care [8,17,34,35]. One approach to fostering greater job satisfaction through thriving at work is by not necessarily removing stressors and demands, but by exploring behaviors around what resources enable individuals to create new ways of working, coping, and developing [13].

Thriving organizations inspire relational and comradery workforces [27]. Comparatively, nurses also look to and require quality peer relationships if they are to accomplish work in professional ways, experience engagement and feel proud of their work [25,34]. The encouragement of positive and collaborative social interactions within healthcare will strengthen the work climate, encourage greater learning opportunities, improve feelings of energy, and enhance wellbeing outcomes, all of which contribute to thriving at work [5,23,37,40]. Nurses want to work around like-minded peers who are encouraging, enthusiastic, and view support as a reciprocated relationship [53].

Teamwork and the promotion of multi-disciplinary teams is well researched within nursing and aligns with the agentic work behavior of thriving, specifically heedful relating, which considers a shared togetherness between colleagues [38,40,54]. Team nursing should be considered for working towards common goals and for the championing of new ideas as a collective, rather than an individual effort [23,49]. Within nursing teams, open communication and breakdowns in the hierarchy are vital for a strong culture, and if individuals are feeling like they are unable to speak up, then the risks of errors and patient safety being undermined increase [54].

Teams and leaders who demonstrate empathy and emotional intelligence with regards to each other’s feelings are deemed essential across the literature, with strong connections linked to thriving at work, commitment, wellbeing, and connectivity [18,19,31]. Nurses are particularly vulnerable when there is a lack of empathy and support, so leaders who demonstrate these skills and qualities will enjoy a workplace where nurses experience less emotional exhaustion [5,55].

A driving foundation for thriving at work, as well as quality nursing and improved patient outcomes, is the relationship and connectivity between employees and their leaders [24,39,48]. Employees deserve to feel safe, valued and that their wellbeing matters, yet these are all documented reasons why nurses intend to leave the workforce [7,8,18,35,38]. Perceived organizational or supervisor support is discussed in both the business and nursing literature, with both stating that when an employee’s contribution is valued, individuals will experience pleasant affective feelings, job satisfaction, a sense of obligation and positive relationships/heedful relating, all of which are associated with thriving at work [7,15,16,17,19].

Recognition and acknowledgement of employees’ work is important for guiding goals, engagement in learning experiences and enhancing positive feelings of vitality [15,17]. Furthermore, low remuneration for nurses is well recognized as triggering feelings relating to a lack of acknowledgement and is inconsistent with effort [56,57]. Feedback is a recognized enabler to thriving and nurses in particular look to feedback for role meaningfulness, original thinking, self-development and motivation [18,39]. When nurses are recognized and praised for their efforts, they are impacted by feelings of happiness, which is described by Zhao et al. [57] as a common variable for individual wellbeing.

As previously mentioned, leaders need to allow for freedom and autonomy in their employees’ ability when making decisions, expressing ideas and exercising choices, in order for work efforts to be smarter, more focused and less energy depleting [19,33,38,40]. A leaderful practice approach allows participation of the team as a collective, grants autonomy and decision-making freedom, and allows nurses to work within their full scope, thus experiencing improved psychological wellbeing and self-development, which leads to thriving at work [13,17,18,50].

By leaders communicating ideas and information transparently and providing an ‘open door policy’, they will inherently fuel thriving and feelings of competence for employees, both in their everyday work and when faced with challenges [15,27,31]. This sharing of information is a contextual feature of thriving at work which encourages bold well-informed problem-solving and shows a degree of acknowledgement from superiors, which is beneficial for productivity and confidence [27,33,37]. From a nursing perspective, managers who restrict how and what information is shared will only impede workflow, yet by communicating proficiently, we know that barriers and inequalities between leaders and employees are reduced [7,35,50].

The organizational literature which discusses thriving at work is based on social exchange theories, whereby relationships are looked on as a balancing act of exchanges based on risks and rewards, culminating in mindfulness and goodwill [17,58]. This is an interesting angle from which to look at interpersonal relationships for nurses, for example, if a nurse is to perceive their work environment as under-resourced with poor support from seniors, they may think that the risks outweigh any benefits, which will increase the risk of absenteeism and turnover [7,41]. Thriving at work outcomes could be influential in this situation, as nurses would be feeling more engaged, trusted, positive, invigorated, and supported to cope with the constantly changing healthcare work environment [7,13,48].

### 4.5. Thriving at Work: Outcomes for Nurse Sustainability and Wellbeing

Thriving is related to better health and wellbeing of staff, and organizations are increasingly focused on providing a positive work life [47]. Therefore, healthcare organizations should focus on the relationship between nurse wellbeing and the delivery of quality care because wellbeing acts “as an antecedent, rather than a consequence of quality care” [43]. The surge in energy and improvement in physical health that employees experience when thriving should be of interest to healthcare organizations because nurses are at greater risk of reporting stress over other expert groups [37,44].

The sustainability of human resources is influenced by the construct of thriving at work due to its relationship with behavioral outcomes and enhanced productivity [37,47]. Nursing, in particular, is facing critical staffing issues, triggering strained working environments in which there is often poor morale [5,41,46]. The strain associated with these stressful environments results in poorer mental health outcomes, but using a thriving at work approach may help mitigate this risk by enhancing energy and self-development [13,18,22,34,44].

Happiness and factors which encourage job satisfaction for nurses need to be emphasized by leaders if sufficient staff levels are to be sustained [18,24]. The more autonomy, trust, and perceived control over one’s work one has, will positively influence vitality, wellbeing, feelings of empowerment, and commitment [5,17,33]. Leaders embracing a relationship-driven leadership style and environment will result in a workplace in which nurses experience positive emotions and thrive at work [18,24,31,37].

The concept of engagement relates well with the positive definition of wellbeing, in that it aims to foster energy and remove the risk of negative effects developing [21,59]. For nursing, a lack of engagement is often related to poor job control and burnout, however by promoting thriving at work (which results in greater job engagement), the vulnerability to burnout and rates of intention to leave should start receding [7,22,33]. Job engagement is also described by Setti and Argentero [21] as “an important protective function for healthcare workers in coping with adversities” (p. 426).

The positive psychology concept of thriving at work offers a different perspective to healthcare organizations than the construct of resilience. Thriving results in high-level performance, development, success and holistic functioning, which simultaneously contributes to improving an individual’s performance and health [33,47]. The social embeddedness of thriving can give individual staff and the wider team the feeling that they are in control despite the chaos and change they experience, reducing the risk of burnout [39,45]. Resilience, on the other hand, is viewed as a combination of physical, psychological, and personality traits which help individuals maintain equilibrium after hardship, rather than a journey of self-development which is the underlying theory of thriving at work [13,57]. So comparatively, although the constructs are similar, thriving (experiencing learning and vitality) can occur whether an individual is faced with adversity or not and is based on positive experiences rather than on behavioral traits [13].

This review was focused on whether the positive organizational scholarship approach underpinning in the Thriving at Work model provides a systematic approach that could be used by health managers to create healthy workforce environments. It therefore did not explore other issues that impact on nurses, such as race, age, and wages. One would expect these issues to be areas of concern that would be addressed when using the Thriving at Work approach.

## 5. Limitations

This was an integrative review, not an exhaustive systematic review. There may be additional relevant literature available. A combination of nursing and non-nursing databases were accessed to provide a wide overview of the topic, and the reviewed literature was limited to articles in English.

## 6. Conclusions

The issues facing the recruitment and retention of nurses in the New Zealand healthcare system and worldwide are becoming critical. Supporting nurses to be highly engaged with and energized by their work so that they do not leave the profession is therefore vital to the long-term effective functioning of health systems. This review provides strong evidence that the Social Embeddedness of Thriving at Work Model offers a very useful framework for developing organizational approaches that support nurses.

Using this model as a framework to develop managerial approaches that support the wellbeing of their nursing workforce because the nurses are thriving at work, healthcare organizations would prioritize the antecedents of thriving at work. Leaders would be transparent, empowering, supportive, allow decision-making freedom, and encourage autonomous behaviors. They would ensure the workforce culture is one of comradery, collaboration, empathy, trust, respect, fairness and mutual support because thriving is directly influenced by the mood of the working environment. For nurses, other ongoing issues such as workload, resources, and safe staffing levels are also crucial. The Thriving at Work framework provides managers with a systematic organizational approach to developing and sustaining a well-functioning healthy nursing workforce.

## Figures and Tables

**Table 1:** Inclusion and exclusion criteria.

Inclusion Criteria	Exclusion Criteria
English language	Community-based
2005–2019	Aged care
Hospital-based nurses	Maternity
Discussion on organizational factors	Student nurses
Human-related studies	Focus on individual factors
Business literature	Discussion on ‘failure to thrive’ rather than ‘thriving at work’
Psychology literature	
Both components (learning and vitality) of thriving at work discussed	

**Table 2:** Summary of findings from research studies.

Reference	Purpose/Aim	Method	Findings	Evidence	Quality
Abid, G., Zahra, I., and Ahmed, A. (2016) [14]. Promoting thriving at work and waning turnover intention: A relational perspective. *Future Business Journal*, *2*(2), 127–137. doi:10.1016/j.fbj.2016.08.001	What are the antecedents of thriving at work and turnover intention? What is the relationship between perceived organizational support and relational resources?	Questionnaire. 128 software developers.	Heedful relating enhances thriving at work. Heedful relating minimizes intention to leave due to connectiveness among individuals. Perceived organizational support positively impacts heedful relating and thriving at work.	3	A
Bensemmane, S., Ohana, M., and Stinglhamber, F. (2018) [32]. Team justice and thriving: a dynamic approach. *Journal of Managerial Psychology*, *33*(2), 229–242. doi:10.1108/JMP-07-2017-0223	Perceived team justice is known to fluctuate within individuals over time, and in response to events at work Is transient team justice predictive of employees transient thriving at work? Is transient self-efficacy an underlying mechanism?	Questionnaire in four waves. 395 individuals completed the first wave, and at least 2 of the 3 subsequent questionnaires. Total 1412 cases. Business master’s students.	Transient overall team justice positively predicts self-efficacy, and therefore thriving at work. Team justice is related to thriving at work. Consideration of how team justice fluctuates is required. Self-efficacy is an important personal resource for team justice and thriving at work. Further mediators need identifying.	3	A
Brown, D., Arnold, R., Fletcher, D., and Standage, M. (2017) [33]. Human thriving. A conceptual Debate and Literature Review. *European Psychologist. 22*(3), 167–179. doi:10.1027/1016-9040/a000294	To investigate key processes that underpin thriving at work. To propose a conceptualization of thriving applicable across different populations and domains. To identify personal and contextual enablers of thriving at work.	Literature Review.	Thriving at work is seen as multifactorial. To achieve thriving at work, subjective individual high-levels of wellbeing and holistic functioning is important. Important to distinguish differences between thriving, prospering, flourishing, growth. Psychosocial variables-personal and contextual enablers. Personal enablers: positive perspective, spirituality, motivation, proactive personality, knowledge and learning, resilience, social competence. Contextual enablers: challenge environment, attachment, trust, family support, colleague and employer support.	5	B
Carmeli, A., and Spreitzer, G. (2009) [23]. Trust, connectivity, and thriving: Implications for innovative behaviors at work. *The Journal of Creative Behavior*, *43*(3), 169–191. doi:10.1002/j.21626057.2009.tb01313.x	Investigating the relationship between trust, connectivity, thriving and innovative behaviors?	Survey completed 3 weeks apart (same survey). Response rate 74.78%. 172 participants from a variety of organizations and industries.	Connectivity mediates the relationship between trust and thriving. Thriving mediates connectivity and innovative behaviors. Viewed as relational and psychological antecedents to how individuals engage in innovative work behaviors. Thriving increases individuals capability to demonstrate innovative, creative and momentum for ideas. Trust is viewed as a psychological contract that augments connectivity/collegial relationships. Results support the hypothesized theoretical model.	3	A
Fiabane, E., Giorgi, I., Sguazzin, C., and Argentero, P. (2013) [34]. Work engagement and occupational stress in nurses and other healthcare workers: the role of organisational and personal factors. *Journal of Clinical Nursing*, *22*(17–18), 2614–2624. doi:10.1111/jocn.12084	To identify the role of organizational and personal factors in predicting work engagement in healthcare workers. Compare work engagement and occupational stress perceptions of healthcare workers.	Cross-sectional survey. 110 hospital staff (registered nurses, nurse aides, physicians, physiotherapists).	Energy predicted by workload, mental health, job satisfaction. Professional efficacy predicted by values and job satisfaction. Physiotherapists had the highest levels of stress and disengagement. Improving psychological health requires a focus on workloads, personal expectations, and job satisfaction.	3	A
Kleine, A.K., Rudolph, C., and Zacher, H. (2019) [15]. Thriving at work: A meta-analysis. *Journal of Organizational Behavior.* 1–27. doi:10.1002/job.2375	Using Spretizer et al.’s (2005) model for thriving at work as a basis, the constructs were investigated and organized into antecedents and outcomes of thriving.	Meta-analysis.	Individual characteristics associated with thriving at work are (a) psychological capital, (b) proactive personality, (c) positive affect, (d) work engagement. Relational characteristics positively associated with thriving at work are (a) supportive co-worker behavior, (b) supportive leadership behavior, (c) perceived organizational support. Thriving at work related to positive employee outcomes e.g., burnout, commitment, and task performance. Analysis supports the Spreitzer et al (2005) model and importance of thriving at work.	3	A
Kowalczuk, K., Krajewska-Kułak, E., and Sobolewski, M. (2017) [35]. The Reciprocal Effect of Psychosocial Aspects on Nurses’ Working Conditions. *Frontiers in Psychology*, *8*, 1386. doi:10.3389/fpsyg.2017.01386	Investigate the correlations between different aspects of nurses’ psychosocial working conditions and what factors affect well-being. Investigate what actions should be taken by management to ensure decent physical and mental conditions.	Questionnaire: Psychosocial aspects of work. 789 inpatient working nurses.	Well-being: Conflict and overload impact negatively. Control of work and cognitive control most strongly correlated. Social, supervisor, and collegial support are positively impacted. Physical well-being correlates with psychological well-being. Expectation of need for change: When work demands are perceived as being high. Low levels of social support were found. Psychosocial risk monitoring and stress prevention programs should be introduced.	3	B
Laschinger, H.K.S., and Finegan, J. (2005) [25]. Empowering nurses for work engagement and health in hospital settings. *Journal of Nursing Administration*, *35*(10), 439–449. doi:10.1097/00005110-200510000-00005	Relationship between empowerment, employee engagement, and physical and mental health outcomes.	Predictive non-experimental design. Random sample of 285 nurses. Test a theoretical model.	Empowerment strongly related to lower levels of burnout, greater work engagement and physical/mental health. Emotional exhaustion linked to overload, lack of reward/recognition, values congruence.	3	B
Li, M., Liu, W., Han, Y., and Zhang, P. (2016) [36]. Linking empowering leadership and change-oriented organizational citizenship behavior: The role of thriving at work and autonomy orientation. *Journal of Organizational Change Management*, *29*(5), 732–750. doi:10.1108/JOCM-02-2015-0032	Based on the theory of thriving at work, what is the link between empowering leadership and change orientated organizational behavior (OCB).	Questionnaires, 2 stages—203 employees. Structured interviews—80 supervisors. Information technology company.	Empowering leadership positively relates to thriving at work. Empowering leadership critically influences change-orientated OCBs. Employees with high autonomy direction were most positively stimulated by empowering leadership. Leaders need to adopt empowering behaviors, and provide suitable settings for thriving to occur.	3	A
Mortier, A.V., Vlerick, P., and Clays, E. 2016) [31]. Authentic leadership and thriving among nurses: the mediating role of empathy. *Journal of Nursing Management*, *24*(3), 357–365. doi: 10.1111/jonm.12329	Examine the relationship between perceived authentic leadership and two dimensions of thriving (learning and vitality).	Questionnaire, cross-sectional design. 360 nurses.	Authentic and empathic leadership enhances both indicators (learning and vitality) of thriving at work. Leadership positively related to vitality. Empathy mediates vitality but not learning.	3	B
Mushtaq, M., Abid, G., Sarwar, K., and Ahmed, S. (2017) [37]. Forging Ahead: How to Thrive at the Modern Workplace. *Iranian Journal of Management Studies, 10*(4). doi: 10.220059/ijms.2017.2355409/672704	Investigation of contextual factors including organizational support, fairness perception, supervisor support, and civility on employee’s thriving at work. Simultaneously, what is the impact of individual characteristics?	Survey questionnaire. Purposive sampling on variety of occupations in service sector organizations: 221 participants.	Proactive personality, civility, fairness perception, organizational support and supervisor support are all antecedents to thriving.	3	A
Nelson, K., Boudrias, J.-S., Brunet, L., Morin, D., Civita, M. De, Savoie, A., and Alderson, M. (2014) [5]. Authentic leadership and psychological well-being at work of nurses: The mediating role of work climate at the individual level of analysis. *Burnout Research, 1*(2), 90–101. doi:10.1016/j.burn.2014.08.001	Further understand the role of authentic leadership and work climate and associated relationship with psychological well-being.	Time-lagged questionnaire. 406 nurses.	Work climate is an important mediator for the relationship between authentic leadership and psychological well-being. Authentic leadership positively impacts work climate and increases psychological well-being.	3	A
Paterson, T.A., Luthans, F., and Jeung, W. (2014) [38]. Thriving at work: Impact of psychological capital and supervisor support. *Journal of Organizational Behavior*, *35*(3), 434–446. doi:10.1002/job.1907	Is thriving at work linked to self-development? What is the relationship between thriving at work and agentic work behaviors (task focus and heedful relating)? The aim was to explore the relationship between psychological capital (PsyCap) and supervisor support climate, with the outcome of thriving at work.	Online survey. 198 employee-supervisor dyads. Variety of part-time management students (full-time employed) and their direct supervisors. Employees completed the survey 1 month prior to supervisors.	Supervisor-rated employee self-development/performance supports Thriving. Agentic behaviors are positively related to thriving. PsyCap and supervisor support positively relates to agentic behaviors. PsyCap supports thriving via task focus. Supervisor support climate and thriving are affected by task focus.	3	B
Porath, C., Spreitzer, G., Gibson, C., and Garnett, F.G. (2012) [39]. Thriving at work: Toward its measurement, construct validation, and theoretical refinement. *Journal of Organizational Behavior*, *33*(2), 250–275. doi: 10.1002/job.756	Study 1: Investigate the construct validity of thriving at work in relation to affect, career orientation, proactive personality, self-evaluations. Study 2: Relationship between thriving and career development initiative, performance, burnout vs. job satisfaction, health, commitment. Study 3: Understanding the contextual embeddedness of thriving.	Study 1: Thriving survey. 175 undergraduates. 410 young professionals. Study 2: Thriving and Burnout Survey. Three samples: 1 = 276 respondents, 2 = 335 respondents, 3 = 136 respondents. Study 3: Thriving survey. 78 respondents (sample 3 in study 2: one month post university program completion).	Study 1: Evidence of convergent and discriminant validity of thriving in relation to hypothesized constructs. Study 2: Thriving positively related to general health, career development initiative, job performance, and leadership effectiveness. Negatively relates to burnout. Study 3: Thriving varies as individuals work life changes. Thriving is related to and varies across work and non-work contexts.	3	A
Sharif, S.P., Ahadzadeh, A.S., and Nia, H.S. (2018) [17]. Mediating role of psychological well-being in the relationship between organizational support and nurses’ outcomes: A cross-sectional study. *Journal of Advanced Nursing*, *74*(4), 887–899. doi: 10.1111/jan.13501	How does psychological well- being mediate the relationship between organizational support for nursing practice, quality of care, and, job satisfaction in the hospital setting?	Cross-sectional survey. 345 hospital-based nurses.	Psychological well-being and organizational support positively relate to quality of care and job satisfaction. Positive perceived organizational support generates favorable well-being, enhancing quality of care and individual outcomes.	3	A
Sia, S.K., and Duari, P. (2018) [40]. Agentic work behaviour and thriving at work: role of decision making authority. *Benchmarking*, *25*(8), 3225-3237. doi:10.1108/BIJ-07-2017-0204	Examining the contribution of agentic work behaviors and decision-making authority (DMA) to thriving at work. Does DMA have a moderating role in the relationship between agentic work behaviors and thriving at work?	Randomized sample. 330 manufacturing companies’ employees. Below the supervisory level.	The three dimensions of agentic work behaviors (task focus, exploration, and heedful relation), positively and directly contribute to thriving at work. Thriving is higher for employees experiencing DMA.	3	A
Spreitzer, G., and Porath, C. (2014) [27]. Self-determination as nutriment for thriving: Building an integrative model of human growth at work. In M. Gagne (Ed.), *The Oxford handbook of work engagement, motivation, and self-determination theory.* doi:10.1093/oxfordhb/9780199794911.016	Discuss key outcomes and antecedents of thriving at work. Focus on self-determination theory and how thriving relates to autonomous motivation.	Book chapter	The three nutriments of autonomous motivation: (1) autonomy, (2) competence, and (3) relatedness, are powerful facilitators of thriving at work. Organizations wanting to promote thriving need to consider autonomy, competence, relatedness enhancement - starting with decision-making, sharing info, creating a culture of community/trust/respect, providing feedback, mitigating volatility to change, and providing flexible work hours. Individual and organizational outcomes of thriving are vast.	5	A
Spreitzer, G., Sutcliffe, K., Dutton, J., Sonenshein, S., and Grant, A.M. (2005) [13]. A socially embedded model of thriving at work. *Organization Science*, *16*(5), 537–549.	Develop a model that explains the social embeddedness of thriving at work. How do work contexts affect individuals?	Model development.	Individuals who self-adapt to psychological states/internal feelings will thrive/undertake change. Organizations need to enable positive participation and well-being. Focus on producing and changing resources. Unit contextual features and resources created in ‘the doing of work’ cultivate agentic working behaviors.	5	A
Utriainen, K., Ala-Mursula, L., and Kyngäs, H. (2015) [18]. Hospital nurses’ wellbeing at work: a theoretical model. *Journal of Nursing Management*, *23*(6), 736–743. doi: 10.1111/jonm.12203	To develop a theoretical model of hospital nurses’ well-being at work.	Model development. Empirical data from 233 nurses.	Themes which support well-being: collegial relationships, enhancing high-quality patient care, supportive and fair leadership, challenging/meaningful and well organized work, opportunities for professional development.	3	A
Verhaeghe, R., Vlerick, P., Gemmel, P., Maele, G. Van, and Backer, G. De. (2006) [41]. Impact of recurrent changes in the work environment on nurses’ psychological well-being and sickness absence. *Journal of Advanced Nursing*, *56*(6), 646–656. doi:10.1111/j.1365-2648.2006.04058.x	How is psychological well-being (job satisfaction, distress) and absence from work impacted by recurrent changes?	Cross-sectional questionnaire. 2094 hospital-based nurses.	Changes in the work environment negatively impact psychological well-being. Distress was high in nurses confronted with threatening changes—Job satisfaction was low, sickness rates were high. Challenging changes were positively related partially to wellbeing (job satisfaction, eustress) but did not change distress or sickness levels.	3	B
